# Possible sleep bruxism, smartphone addiction and sleep quality among Brazilian university students during COVID-19 pandemic

**DOI:** 10.5935/1984-0063.20220036

**Published:** 2022

**Authors:** Ivana Meyer Prado, Matheus de França Perazzo, Lucas Guimarães Abreu, Ana Flávia Granville-Garcia, Maryam Amin, Isabela Almeida Pordeus, Saul Martins Paiva, Junia Maria Serra-Negra

**Affiliations:** 1 Universidade Federal de Minas Gerais, Department of Pediatric Dentistry - Belo Horizonte - Minas Gerais - Brazil.; 2 Universidade Estadual da Paraíba, Department of Dentistry - Campina Grande - Paraíba - Brazil.; 3 University of Alberta, Division of Pediatric Dentistry - Edmonton - Alberta - Canada.

**Keywords:** Pandemics, Sleep Bruxism, Sleep, Smartphone, Students

## Abstract

**Objectives:**

To evaluate the association of sleep bruxism activity with smartphone addiction and sleep quality among university students during COVID-19 pandemic.

**Material and Methods:**

A cross-sectional online survey with 546 university students in social distancing was conducted (May 29^th^ to June 2^nd^ 2020). Participants should be undergraduate and graduate students enrolled in Brazilian public/private universities. A self-completed questionnaire collected sociodemographic characteristics, academic information, and severity of possible sleep bruxism (PSB) activities (grinding, bracing, and thrusting). Students answered the Brazilian version of Pittsburgh sleep quality index (PSQI-BR) and short form of the smartphone addiction scale (SAS-SV). Descriptive statistics and multinomial logistic regression were performed (*p*=0.05).

**Results:**

Sample mean age was 24.9 (±5.5) years. Students with higher scores of PSQI-BR were more likely to present severe PSB-bracing (OR=1.154; 95%CI=1.057-1.260), severe PSB-grinding (OR=1.133; 95%CI=1.048-1.225) and severe PSB-thrusting (OR=1.197;95%CI=1.107-1.294). Students who had children presented 3 times more chance (OR=3.193; 95%CI=1.236-8.250) to report severe PSB-thrusting. Being female increased the chance of reporting moderate (OR=3.315; 95%CI=1.333-8.914) and severe (OR=2.940; 95%CI=1.116-7.747) PSB-thrusting. Students not enrolled in distance learning presented 2 times more chance (OR=2.638; 95%CI=1.233-5.649) of reporting moderate PSB-grinding. Students with higher scores in SAS-SV had a slight increase in the chance of presenting mild (OR=1.042; 95%CI=1.009-1.077) and moderate (OR=1.065; 95%CI=1.018-1.115) PSB-bracing, as well as mild (OR=1.044; 95%CI=1.011-1.078) and moderate (OR=1.041; 95%CI=1.005-1.077) PSB-thrusting.

**Conclusion:**

Smartphone addiction, worse sleep quality, having children, female sex and not being enrolled in distance learning were associated possible sleep bruxism during COVID-19 pandemic.

## INTRODUCTION

COVID-19 has become a global disease and due to the lack of specific treatments or vaccines, preventive measures, including social distancing and quarantine, were the first strategies to mitigate the spread of the virus SARS-CoV-2^1^. As a consequence, people have faced new emotional challenges involving stress, uncertainty and fear^2^. Their lives have suddenly changed in a drastic, surprising and difficult way^2^. A sense of being stuck, lack of control, helplessness, uncertainty about the future, and feelings of frustration, worry and anxiety have been reported by participants in studies during the COVID-19 pandemic^2^. Those emotional reactions could influence and interfere with individuals’ health, well-being, quality of life and sleep^3^, impacting on sleep bruxism behavior^4^.

Sleep and awake bruxism are usually considered different behaviors, with different definitions^5^. Sleep bruxism is a masticatory muscle activity during sleep, characterized as rhythmic (phasic) or non-rhythmic (tonic), while awake bruxism is a masticatory muscle activity that occurs during wakefulness, characterized by repetitive or sustained tooth contact and/or by bracing or thrusting of the mandible^5^. Based on the tools used for diagnosis, bruxism may be defined as: ‘possible’ bruxism, diagnosed based on a positive self-report; ‘probable’ bruxism, diagnosed based on a positive clinical inspection with or without a positive self-report; and ‘definite’ bruxism, diagnosed based on a positive instrumental assessment with or without a positive self-report and/or a positive clinical inspection^5^. Bruxism muscle activity can be characterized by clenching or grinding of the teeth and or by bracing or thrusting of the mandible^5^, with bracing meaning forcefully maintaining a certain mandibular position and thrusting meaning forcefully moving the mandible in a forward or lateral direction. Both activities would not necessarily involve tooth contact^5^. Sleep bruxism is a behavior regulated centrally^5^, with a multifactorial etiology, strongly associated with emotional and behavioral factors such as, stress, smoking, alcohol use and caffeine consumption, as well as sleep disorders and sleep quality^4,6,7^.

Staying confined at home while working and studying might increase the use of internet and smartphones. Modern smartphones are not only used to make calls, but are also used as a computer, media player and video camera, providing information anytime, anywhere^8,9^. Smartphones can make people’s lives more convenient, but they can also become a social issue, with far-reaching negative effects on daily activities^9^. The use of electronic media and the device’s blue light may have a negative impact on sleep and shorten the total sleep time^10-12^. As a result, sleep deprivation and interferences on the circadian cycle can negatively impact mental, social, and physical health^12^. The sleep disturbance associated to electronic device use at night may be a partial mediator of depressive symptoms^13^. Moreover, during a pandemic, such as the one caused by COVID-19, young people might be using the smartphone for a longer period of time and consequently having sleep and emotional problems.

Due to the COVID-19 pandemic, the Brazilian government declared a state of emergency, forcing everybody to stay at home and to adhere to social distancing. Changes in daily routines, habits, anxiety related to the exposure to a new disease and social isolation might interfere in the pattern of smartphone use, sleep quality, and bruxism activity. Psychological factors related to the COVID-19 pandemic could lead to a greater risk of developing or worsening bruxism behavior^14^. Therefore, the present study aimed to evaluate the association of possible sleep bruxism (PSB) with smartphone addiction and sleep quality among undergraduate and graduate Brazilian students during social distancing mandates issued because of the COVID-19 pandemic. The study hypothesis is that smartphone addiction and poor sleep quality were associated to possible sleep bruxism activity during COVID-19 pandemic.

## MATERIAL AND METHODS

### Study design, setting, and participants

A cross-sectional online open survey^15^ was conducted with undergraduate and graduate students enrolled in Brazilian public and private universities. Data were collected during the COVID-19 pandemic (from May 29 to June 02, 2020), by means of a snowball sampling^16^ through an online questionnaire available on Google Forms platform (Google Inc., Menlo Park, CA, USA). The link to the questionnaire was sent via WhatsApp (WhatsApp Inc., Mountain View, CA, USA) and via email messages forwarded to undergraduate and graduate students attending Brazilian universities. Students entered manually into the platform, by clicking the link. Despite the limitations of epidemiological data collection imposed by the pandemic, evidence shows that data results originating from paper-and-pencil and computerized survey models are equivalent^17^. Participants spent approximately 10 minutes to complete the questionnaires.

Undergraduate and graduate students enrolled in Brazilian public and private universities were included and they digitally consented their participation through an informed consent form and answered all questions from the online questionnaire. Shortly before the ethical considerations form, they answered questions about their systemic conditions. Those reporting syndromes, cognitive disorders or use of anticonvulsant medication were excluded^18^. Information regarding students’ health was provided by participants.

### Ethical considerations

This study was approved by the ethics and human research committee of *Universidade Federal de Minas Gerais* (protocol #33872020.5.0000.5149) and was conducted according to the set of principles stated in the declaration of Helsinki (revised in World Medical Association, 2013).

### Data collection

Researchers contacted associations and collegiate boards from Brazilian universities and asked professors to share the questionnaire link with their students. Students answered an online questionnaire with five pages. After answering the questions, respondents were able to review and change their answers, if necessary. First page presented the informed consent form. After agreeing to participate, participants were directed to the next page, with information whether they were practicing social distancing, their sociodemographic characteristics, their formation level and study area, type of educational institution they were enrolled in and if they were enrolled in distance learning during social distancing. Third page of the questionnaire had questions about possible sleep bruxism (PSB) activities (grinding, bracing and thrusting).

The frequency of PSB activities (grinding, bracing and thrusting) was evaluated by means of three questions with a recall of what had occurred in the previous month. Questions were based on previous studies^5,19,20^. The questions were as follows:

1. Sleep bruxism grinding activity - in the past month, did you notice, or someone told you, that you grind your teeth during sleep?
2. Sleep bruxism thrusting activity - in the past month, when waking up in the morning or during the night, have you had your jaw positioned forward or sideways?
3. Sleep bruxism bracing activity - in the past month, when waking up in the morning or during the night, have you had your jaw in a steady/rigid position (with difficulty in opening your mouth)?

All questions could be answered with “none in the past month”, “less than once a week”, “once or twice a week” and “three or more times a week”^21^. PSB activities were categorized as: “absent” if the behavior had not taken place in the previous month; “mild” if the behavior had taken place less than once a week in the previous month; “moderate” if the behavior had taken place once or twice a week in the previous month; “severe” if the behavior had taken place three or more times a week in the previous month^21^.

The Brazilian Portuguese version of the Pittsburgh sleep quality index (PSQI-BR) was used in this study in the fourth page of the online questionnaire^22^. The original Pittsburgh sleep quality index (PSQI) was developed to assess sleep quality in the past 30 days through a standardized questionnaire^23^. It contains 19 self-rated questions, used to calculate PSQI global score, and five questions rated according to the information gotten from a roommate or a bed partner, and were used for clinical purposes^22,23^. The PSQI has seven components (subjective sleep quality, sleep latency, sleep duration, habitual sleep efficiency, sleep disturbances, use of sleep medication, daytime dysfunction), each one scored equally on a 0-3 scale. Later, the seven components are summed up to yield a global PSQI score, ranging from 0 to 21. The highest score indicates the worst sleep quality^22,23^.

Participants also answered the Brazilian Portuguese short version of the Smartphone Addiction Scale (SAS-SV) in the fifth page of the questionnaire^9,24^. This instrument contains 10 questions with which smartphone addiction among adolescents is evaluated^9,24^. The questions can be answered with a six-point Likert scale (1 = “strongly disagree”; 2 = “disagree”; 3 = “slightly disagree”; 4 = “slightly agree”; 5 = “agree”; and 6 = “strongly agree”). The score varies between 10 and 60. A higher score indicates higher levels of smartphone addiction^9,24^.

### Pilot study

Prior to the main study, a pilot study was carried out involving 10 undergraduate students in order to evaluate the proposed methods. Participants from the pilot study were not included in the main study. After answering the questionnaire, participants of the pilot study could provide suggestions and comments about the methodology. Based on their response, no change was needed to the proposed methods.

### Statistical analysis

Data were analyzed using the Statistical Package for the Social Sciences (SPSS for Windows, version 21.0, SPSS Inc., Chicago, Illinois, USA). Descriptive statistics, bivariate and multivariate multinomial logistic regression were performed to evaluate the association of trait EI, smartphone addiction, sleep quality, academic information and activities (e.g., degree level and if student is enrolled in distance learning) with PAB and PSB severity. All variables with *p*-value<0.20 for the association with PAB and PSB in the bivariate analysis were incorporated into the multivariate regression model. The results of multinomial analyses were provided in terms of odds ratio (OR), confidence interval (CI) and *p*-values, with statistical significance level set at 5.0% (*p*-values<0.05).

## RESULTS

A total of 564 students answered the questionnaire. Among those, 546 individuals were included in the study (96.8% completion rate) and 18 (3.2%) were excluded because they were unable to practice social distancing during the COVID-19 pandemic. Most participants were from the southeast (52.7%) and northeast (37.0%) regions of Brazil, followed by mid-west (4.4%), north (2.9%) and south (2.9%) regions. Students’ mean age was 24.9 (±5.5) years old. Most participants were female (74.5%), undergraduate students (69.1%) and had no children (91.8%). PSB - grinding activity was reported by 21.6% of participants, among whom 8.6% had severe activity, 7.1% had moderate activity and 5.9% had mild activity. The prevalence of all activities of PSB is presented in Table 1. Mean PSQI-BR global score was 7.8 (±3.8), ranging from 1 to 20 (Table 2).

**Table 1:** Descriptive analysis of sociodemographic characteristics, degree level, smartphone addiction and sleep bruxism among university students from Brazil under social distancing during COVID-19 pandemic in 2020.

Variables	Frequency (%)
**Age** Mean [±SD]	24.9 [±5.5]
Median [Min.-Max.]	24.0 [18-58]
**With children** Yes	45 (08.2)
No	502 (91.8)
**Sex** Female	407 (74.4)
Male	139 (25.4)
Non-binary	01 (0.2)
**Degree level** Graduate	378 (69.1)
Undergraduate	169 (30.9)
**Distance learning** Yes	243 (44.4)
No	304 (55.6)
**SAS-SV** Mean [±SD]	31.8 [±9.7]
Median [Min.-Max.]	32.0 [10-58]
Addicted	245 (44.9)
Not addicted	301 (55.1)
**PSQI-BR global score** Good sleep quality (score<5)	308 (56.3)
Poor sleep quality (score>5)	239 (78.8)
**Possible SB - bracing activity** Absent	435 (79.5)
Mild	49 (09.0)
Moderate	27 (04.9)
Severe	36 (06.6)
**Possible SB - grinding activity** Absent	429 (78.4)
Mild	32 (05.9)
Moderate	39 (07.1)
Severe	47 (08.6)
**Possible SB - thrusting activity** Absent	405 (74.0)
Mild	51 (09.3)
Moderate	42 (07.7)
Severe	49 (09.0)

Abbreviation: SD = Standard deviation; Min. = Minimum; Max. = Maximum; SB = Sleep bruxism.

**Table 2:** Descriptive analysis of scores and components of the Brazilian Portuguese version of Pittsburg sleep quality index (PSQI-BR) among university students from Brazil under social distancing during COVID-19 pandemic in 2020.

PSQI-BR	Mean (±SD)	Median [Min. - Max.]	Score 0		Score 1		Score 2		Score 3	
			N	%	N	%	N	%	N	%
Subjective sleep quality	1.37 (±0.7)	1.0 [0 - 3]	57	10.4	279	51.0	162	29.6	49	09.0
Sleep latency	1.6 (±1.0)	2.0 [0 - 3]	78	14.3	180	32.9	129	23.6	160	29.3
Sleep duration	0.6 (±0.9)	0.0 [0 - 3]	324	59.2	103	18.8	85	15.5	35	06.4
Habitual sleep efficiency	0.6 (±0.9)	0.0 [0 - 3]	327	59.8	120	21.9	52	09.5	48	08.8
Sleep disturbances	1.4 (±0.6)	1.0 [0 - 3]	12	02.2	291	53.2	212	38.8	32	05.9
Use of sleeping medication	0.4 (0.8)	0.0 [0 - 3]	424	77.5	51	09.3	33	06.0	39	07.1
Daytime dysfunction	1.5 (±0.7)	2.0 [0 - 3]	47	08.6	205	37.5	239	43.7	56	10.2
Global score	7.8 (±3.8)	7.0 [1 - 20]	-	-	-	-	-	-	-	-

Abbreviation: PSQI-BR = Brazilian Portuguese version of Pittsburg sleep quality index; SD = Standard deviation; Min. = Minimum; Max. = Maximum.

Table 3 displays the unadjusted and adjusted multinomial logistic regression model for PSB - bracing activity. The adjusted model showed that female students (Odds Ratio [OR] = 2.393; 95% Confidence Interval [CI] = 1.037-5.522; probability value [*p*]=0.041) and students with a higher score in SAS-SV (OR=1.042; 95%CI=1.009-1.077; *p*=0.014) were more likely to present mild PSB - bracing activity. Students with higher scores in SAS-SV (OR=1.065; 95%CI=1.018-1.115; *p*=0.007) were also more likely to present moderate PSB - bracing activity. Severe PSB - bracing activity was associated with student’s age (OR=1.057; 95%CI=1.001-1.117; *p*=0.048), female sex (OR=12.957; 95%CI=1.735-96.762; *p*=0.013) and higher scores of PSQI-BR (OR=1.154; 95%CI=1.057-1.260; *p*=0.001).

**Table 3:** Non-adjusted and adjusted multinomial logistic regression evaluating sleep bruxism - bracing activity severity and sociodemographic characteristics, sleep quality and smartphone addiction among university students from Brazil under social distancing during COVID-19 pandemic in 2020.

Sleep bruxism - bracing activity												
	Mild				Moderate				Severe			
Variables	Non-adjusted model OR (95%CI)	*p*	Adjusted model OR (95%CI)	*p*	Non-adjusted model OR (95%CI)	*p*	Adjusted model OR (95%CI)	*p*	Non-adjusted model OR (95%CI)	*p*	Adjusted model OR (95%CI)	*p*
**Age**	0.969 (0.908-1.033)	.333	0.986 (0.923-1.053)	.673	0.984 (0.910-1.065)	.693	1.017 (0.938-1.103)	.680	1.053 (1.005-1.103)	**.029**	1.057 (1.001-1.117)	**.048**
**With children**												
Yes	0.486 (0.113-2.087)	.332			1.429 (0.410-4.981)	.576			1.843 (0.674-5.040)	.233		
No	1				1				1			
**Sex**												**.013**
Female	2.392 (1.047-5.469)	**.039**	2.393 (1.037-5.522)	**.041**	1.082 (0.444-2.639)	.862	1.171 (0.471-2.911)	.734	13.955 (1.891-102.974)	**.010**	12.957 (1.735-96.762)	
Male	1		1		1		1		1		1	
**Degree level**												
Graduate	0.991 (0.522-1.881)	.978			0.786 (0.325-1.904)	.594			1.270 (0.624-2.582)	.510		
Undergraduate	1				1				1			
**Distance learning**												.630
No	1.189 (0.652-2.167)	.572	1.324 (0.716-2.450)	.369	2.343 (0.971-5.656)	.058	2.457 (0.996-6.250)	.051	0.656 (0.331-1.300)	.227	0.838 (0.409-1.715)	
Yes	1		1		1		1		1		1	
**PSQI-BR global score**	1.030 (0.953-1.113)	.462	1.000 (0.917-1.089)	.992	1.017 (0.917-1.128)	.745	0.984 (0.875-1.107)	.791	1.186 (1.091-1.290)	**<.001**	1.154 (1.057-1.260)	**.001**
**SAS-SV**	1.043 (1.011-1.075)	**.007**	1.042 (1.009-1.077)	**.014**	1.055 (1.013-1.098)	.010	1.065 (1.018-1.115)	**.007**	1.017 (0.982-1.053)	.348	1.013 (0.975-1.051)	.515

Abbreviations: *p* = Probability value; OR = Odds ratio; CI = Confidence interval; TEIQue-SF = Traits of emotional intelligence questionnaire - short form; SAS-SV = Smartphone addiction scale - short version. Values in bold represent statistically significant associations (*p*<0.05).

Table 4 shows the adjusted model evaluating PSB - grinding activity severity and assessed variables. The adjusted model demonstrated that students who were not enrolled in distance learning were more likely to report moderate PSB - grinding activity (OR=2.638; 95%CI=1.233-5.649; *p*=0.012). Students with higher scores of PSQI-BR were more likely to present severe PSB - grinding activity (OR=1.133; 95%CI=1.048-1.225; *p*=0.002).

**Table 4:** Non-adjusted and adjusted multinomial logistic regression evaluating sleep bruxism - grinding activity severity and sociodemographic characteristics, sleep quality and smartphone addiction among university students from Brazil under social distancing during COVID-19 pandemic in 2020.

Sleep bruxism - bracing activity												
	Mild				Moderate				Severe			
Variables	Non-adjusted model OR (95%CI)	*p*	Adjusted model OR (95%CI)	*p*	Non-adjusted model OR (95%CI)	*p*	Adjusted model OR (95%CI)	*p*	Non-adjusted model OR (95%CI)	*p*	Adjusted model OR (95%CI)	*p*
**Age**	0.986 (0.917-1.060)	.706	1.010 (0.932-1.095)	.800	1.002 (0.943-1.065)	.950	1.023 (0.953-1.159)	.244	1.044 (0.999-1.091)	.056	1.026 (0.970-1.086)	.372
**With children**												
Yes	0.387 (0.051-2.926)	.358	0.342 (0.040-2.958)		1.000 (0.292-3.422)	1.000	0.910 (0.227-3.648)	.895	2.462 (1.063-5.699)	**.035**	1.834 (0.663-5.077)	.243
No	1		1		1		1		1			
**Sex**												
Female	1.644 (0.660-4.095)	.286	1.633 (0.647-4.120)	.299	1.680 (0.720-3.920)	.230	1.726 (0.729-4.087)	.734	1.850 (0.840-4.074)	1.27	1.621 (0.724-3.631)	.240
Male	1		1		1		1		1		1	
**Degree level**												
Graduate	1.153 (0.541-2.460)	.712			0.759 (0.360-1.603)	.470			0.934 (0.484-1.803)	.839		
Undergraduate	1				1				1			
**Distance learning**												
No	0.559 (0.269-1.161)	.119	0.570 (0.272-1.197)	.138	2.369 (1.127-5.000)	**.023**	2.638 (1.233-5.649)	**.012**	1.012 (0.552-1.855)	.968	1.262 (0.672-2.369)	.468
Yes	1		1		1		1		1		1	
**PSQI-BR global score**	0.977 (0.884-1.808)	.645	0.951 (0.855-1.057)	.347	1.064 (0.979-1.157)	.146	1.057 (0.963-1.159)	.244	1.141 (1.059-1.229)	**.001**	1.133 (1.048-1.225)	**.002**
**SAS-SV**	1.021 (0.984-1.059)	.277	1.022 (0.982-1.064)	.286	1.031 (0.996-1.066)	.080	1.032 (0.995-1.070)	.092	1.009 (0.978-1.041)	.567	1.004 (0.972-1.037)	.790

Abbreviations: *p* = Probability value; OR = Odds ratio; CI = Confidence interval; TEIQue-SF = Traits of emotional intelligence questionnaire - short form; SAS-SV = Smartphone addiction scale - short version. Values in bold represent statistically significant associations (*p*<0.05).

Mild PSB - thrusting activity was associated with being a graduate student (OR=2.433; 95%CI=1.319-4.487; *p*=0.004) and higher scores of SAS-SV (OR=1.044; 95%CI=1.011-1.078; *p*=0.008). Students who had children (OR=3.051; 95%CI=1.084-8.590; *p*=0.035), female students (OR=3.315; 95%CI=1.1459.602; *p*=0.027) and students with higher scores of SAS-SV (OR=1.041; 95%CI=1.005-1.077; *p*=0.023) were more likely to report moderate PSB - thrusting activity. Severe PSB - thrusting activity was associated with having children (OR=3.193; 95%CI=1.236-8.250; *p*=0.017), female sex (OR=2.940; 95%CI=1.116-7.747; *p*=0.029) and higher scores of PSQI-BR (OR=1.197; 95%CI=1.107-1.294; *p*<0.001) (Table 5).

**Table 5:** Non-adjusted and adjusted multinomial logistic regression evaluating sleep bruxism - thrusting activity severity and sociodemographic characteristics, sleep quality and smartphone addiction among university students from Brazil under social distancing during COVID-19 pandemic in 2020.

Sleep bruxism - bracing activity												
	Mild				Moderate				Severe			
Variables	Non-adjusted model OR (95%CI)	*p*	Adjusted model OR (95%CI)	*p*	Non-adjusted model OR (95%CI)	*p*	Adjusted model OR (95%CI)	*p*	Non-adjusted model OR (95%CI)	*p*	Adjusted model OR (95%CI)	*p*
**Age**	0.996 (0.944-1.052)	.897			0.990 (0.931-1.053)	.749			1.024 (0.977-1.074)	.317		
**With children**												
Yes	1.584 (0.580-4.328)	.369	1.287 (0.449-3.685)	.639	2.427 (0.938-6.290)	**.067**	3.051 (1.084-8.590)	**.035**	2.844 (1.209-6.691)	**.017**	3.193 (1.236-8.250)	**.017**
No	1		1		1		1		1		1	
**Sex**												
Female	0.857 (0.456-1.609)	.631	0.769 (0.402-1.473)	.429	3.624 (1.263- 10.398)	**.017**	3.315 (1.145-9.602)	**.027**	3.447 (1.333-8.914)	**.011**	2.940 (1.116-7.747)	**.029**
Male	1		1		1		1		1		1	
**Degree level**												
Graduate	2.499 (1.387-4.505)	**.002**	2.433 (1.319-4.487)	**.004**	0.853 (0.415-1.753)	.665	0.683 (0.314-1.495)	.341	0.868 (0.444-1.695)	.678	0.647 (0.312-1.342)	.242
Undergraduate	1		1		1		1		1		1	
**Distance learning**												
No	1.467 (0.799-2.691)	.216			0.727 (0.385-1.374)	.327			0.904 (0.499-1.638)	.740		
Yes	1				1				1			
**PSQI-BR global score**	1.083 (1.004-1.169)	**.039**	1.052 (0.970-1.140)	.222	1.098 (1.012-1.192)	**.025**	1.074 (0.983-1.173)	.114	1.211 (1.123-1.306)	**<.001**	1.197 (1.107-1.294)	**<.001**
**SAS-SV**	1.047 (1.016-1.080)	**.003**	1.044 (1.011-1.078)	.008	1.043 (1.009-1.078)	**.013**	1.041 (1.005-1.077)	**.023**	1.025 (0.994-1.057)	.108	1.016 (0.985-1.049)	.312

Abbreviations: *p* = Probability value; OR = Odds ratio; CI = Confidence interval; TEIQue-SF = Traits of emotional intelligence questionnaire - short form; SAS-SV = Smartphone addiction scale - short version. Values in bold represent statistically significant associations (*p*<0.05).

## DISCUSSION

As the COVID-19 pandemic unfolded, several changes in lifestyle have taken place^2,25-27^. The increase in screen-time ranged from 65% to 74%^27,28^, being more frequent among young adults^26^, probably due to distance learning and work. Devices’ blue light can interfere on individuals’ sleep^11^ and circadian physiology, negatively impacting on their mental, social, and physical health^12^. Excessive use of smartphones can increase the risk of poor sleep quality, depression, and anxiety^29^, and its use, specially around bedtime, was associated with sleep disturbances, sleep latency, sleep efficiency and daytime dysfunction^30,31^. The present study found that students with higher scores in SAS-AD presented a slight increase in the odds of presenting mild and moderate PSB - bracing and thrusting activities. The characteristics of addiction involved in devices use^8,32,33^, along with its impact on sleep^30,31^ could lead to sleep bruxism behavior^14^, specially bracing and thrusting activity, which are related to muscle activity and not necessarily involving tooth contact^5^. Results of the current study should be considered with caution given that the increasing use of smartphone during COVID-19 pandemic^27,28^ could have occurred as a necessity (remote work and learning, distraction and connecting with family and friends due to physical distancing), instead of an addiction behavior. The validated instrument used in the study was developed to be applied under normal circumstances, and future research after COVID-19 pandemic is encouraged to understand the mechanisms involved in this association.

The non-association of smartphone addiction with severe PSB could be explained by the strong association of higher scores of PSQI and severe PSB. Possibly, sleep quality and sleep disturbances are determinant factors related to severe activity of sleep bruxism, eliminating the influence of smartphone addiction in severe cases. A longitudinal study with children and adolescents found that sleep bruxism was not predictive of internet addiction and internet addiction was not predictive of sleep bruxism behavior^34^. Although, authors found that dyssomnia sequentially predicted internet addiction and internet addiction predicted disturbed circadian rhythm^34^, they believe that children and adolescents with dyssomnias might use internet while they struggle to sleep, but this behavior can cause circadian rhythm disturbances^34^. Those finding reinforce the complexity of the interactions among sleep problems, sleep bruxism and smartphone addiction. Longitudinal studies to evaluate the bidirectional relationships between sleep disorders, sleep bruxism and smartphone addiction are encouraged to clarify the results found.

Good sleep quality is essential for individuals’ health and well-being and several aspects of sleep have been associated with sleep bruxism previously^7,35^, but the association of sleep quality with sleep bruxism severity is a novelty. In previous studies employing polysomnography, sleep bruxism occurred after microarousal episodes during sleep^35^ and microarousal frequency was higher in patients with higher PSQI scores^36^, which could explain the link between sleep quality and sleep bruxism. A Brazilian study found that 55.3% of adults had sleep problems during the COVID-19 pandemic^37^, which could lead to a worsening of sleep bruxism behavior during this period, explaining the association only with severe activity of PSB. Social distancing measures during a pandemic scenario can make people feel safer, but it can affect their sleep, increasing sleep problems^38^, stress level and emotional reactions^2^, which are associated with sleep quality^3^ and sleep bruxism^4^.

Due to COVID-19 pandemic, schools were closed down and children stayed at home, causing disruptions in the families routine^2,39^. Parents reported exhaustion due to excessive task performing and physical and emotional burden^2^. During the pandemic, parents also reported an increase in their screen-time, a reduction of sleep duration, and moderate to high levels of stress caused by financial issues^28^. In the current sample, students who had children were more likely to report moderate and severe PSB - bracing activity. Moderate and severe masticatory muscle activity could be caused by the impact on parents’ sleep, tough changes of habits, and disruption of families’ daily routine. When evaluating the sample’s mean age, it is likely that participants who were parents had young children who demanded more care and attention. Supportive attention should be delivered to young adults with children^28^, and future research is needed to explore behavioral, psychological, and emotional problems experienced by this population and its implication on sleep bruxism behavior.

Being female was also associated with PSB - thrusting and bracing activities. The association between bruxism activity and sex is still controversial. A systematic review evaluating the epidemiology of bruxism showed no association between the behavior and sex^40^, but a recent study demonstrated a slightly higher proportion of male students who ground their teeth^41^. Another recent study found otherwise, self-reported sleep bruxism activity was more frequent among women^42^. The lack of standardized diagnosis methods for bruxism evaluation^6^, and the non-differentiation between awake and sleep bruxism, may have contributed to the controversy among different studies. During the COVID-19 pandemic, women who presented higher emotional reactions and psychiatric symptoms were more likely to present depression and anxiety, besides higher levels of fear and psychological distress when compared to men^2,43,44^. Moreover, women might have increased their domestic workload on a daily basis^45,46^, which could have disrupted their routine and sleep, specially those with children. This could justify the higher prevalence of women with moderate and severe sleep bruxism activity during the pandemic.

Current evidence states that bruxism decreases with age^40^, but as mentioned before, most studies lack standardized diagnosis methods for bruxism, and do not differentiate awake and sleep bruxism^6^. In the current sample, the increase of one year of age represented a slight increase in the odds of reporting severe PSB - bracing activity. Herein, most participants were young adults, with a mean age of 24.9 years, ranging from 18 to 58. Another study with university students found a slight difference of age, with older students presenting a higher prevalence of sleep bruxism^42^. Graduate students were also more likely to report mild PSB - thrusting activity. The associations between bruxism and age and educational degree level could be justified by the fact that older students and graduate students possibly have more responsibilities as adults and might be facing higher levels of stress^44^. Those factors, along with uncertainties about their future, graduation and job opportunities during and after the pandemic, could impact their stress level^4^, sleep^3^ and daily routine^26^, leading to sleep bruxism - bracing and thrusting activities.

In Brazil, universities fully lifted classes and activities on campus at the beginning of the pandemic, then, distance learning was the alternative for several institutions. However, previous preparation and training of students, staff, and professors to deal with electronic activities was scarce^44,47^. Not being enrolled in distance learning was associated with moderate PSB - grinding activity. Some Brazilian institutions suspended on-site classes and were not able to implement distance learning immediately, leaving students without any classes during social distancing. It is important to state that data collection was performed when there was an ascending curve of cases in Brazil, without the perspective of a vaccine or of an end to the pandemic. The uncertainties regarding their future could contribute to an increase on stress and anxiety levels, interfering with their sleep. Moreover, with classes fully lifted and no distance learning activities, students’ daily routine might have been disrupted, changing their sleep hours, habits, and impacting their sleep quality^26,27^. With the extension of the pandemic, distance learning remained the alternative to several institutions in Brazil and a close attention by health care providers to how students respond to learning and routine changes is encouraged to minimize possible impacts on their health and quality of life.

Some limitations of the study are important to be addressed. Due to social distancing mandates, data collection was performed online, which is an equivalent alternative of data collection compared to paper-and-pencil surveys^17^. Students were invited through WhatsApp and email messages containing the link of the questionnaire. In the snowball sampling method, initial subjects are recruited, and those subject recruit other subjects, in a way that the sample expands as a snowball^16^. Unfortunately, in this sampling method, researchers have no control of the number of people presented with a questionnaire^15^. Also, participants who answered the questionnaire might not represent the entire population of Brazilian university students, characterizing a possible selection bias. For that matter, future paper-and-pencil studies with more controlled selection criteria after the pandemic should be performed to confirm the results in other populations. Clinical evaluation of participants was impossible at the time, and, for that matter, the diagnosis of sleep bruxism was based on participants’ self-report^5^. Brazil has been severely hit by the pandemic, and cases and deaths are still on the rise, with several variants in circulation^48^, making clinical evaluation of individuals difficult even now. Polysomnography recordings are the gold standard for the evaluation of sleep bruxism, but it has high costs and limited availability for epidemiological studies^5,8^. Despite this limitation, the evaluation of the different sleep bruxism activities (grinding, bracing, and thrusting) and the severity of this condition based on the frequency of occurrence in accordance with recent consensus was feasible^5^.

Longitudinal studies evaluating the future consequences of COVID-19 pandemic on students’ health, well-being and bruxism activity are also important. Current data was collected when COVID-19 cases were ascending in Brazil, and the population were confined at home, which could interfere on their health and emotional reactions at the time. Further investigation is still needed to understand the impact of the confinement on sleep and health^38^. During the COVID-19 pandemic, individuals presented increasing feelings of frustration, being stuck, lack of control and helplessness^2^, as well as levels of anxiety, stress and depression symptoms^34,37^ which impact their sleep quality^3^. Awareness of associated factors of different bruxism activities (grinding, bracing, and thrusting) severity can help health care professionals understand when and why bruxism activity becomes a harmful behavior. This study represents a great step towards a deeper understanding about sleep bruxism epidemiology, specially under an adverse circumstance such as the COVID-19 pandemic period, whose severity and proportion has no precedent in human history. The results of the current study emphasize the need for a deep understanding of the long-term consequences and implications of COVID-19 on individuals’ health and well-being, as well as the impact of sleep quality, sex, age, smartphone use, distance learning, and parental status on sleep bruxism activity and severity. Bruxism is a complex behavior with a multifactorial etiology^6,3^, and particular attention by oral health care providers to young adults with bruxism is important to minimize the impact and consequences of this condition during the pandemic period and afterwards.
